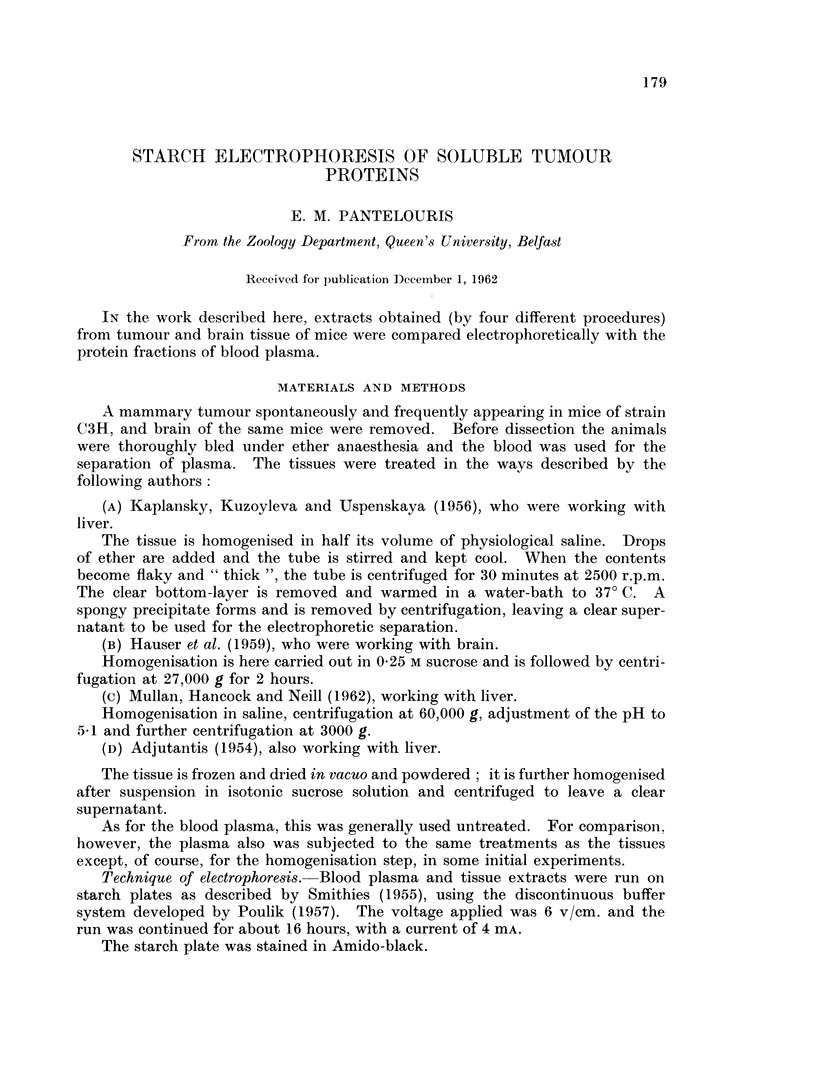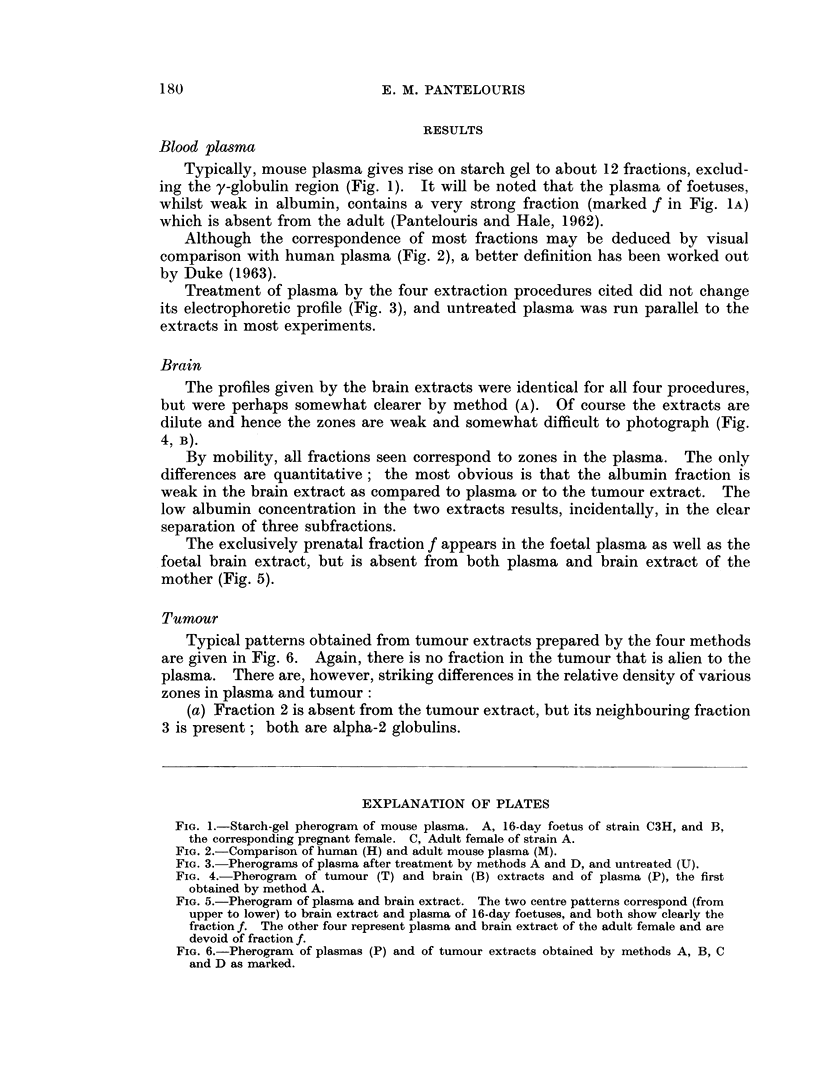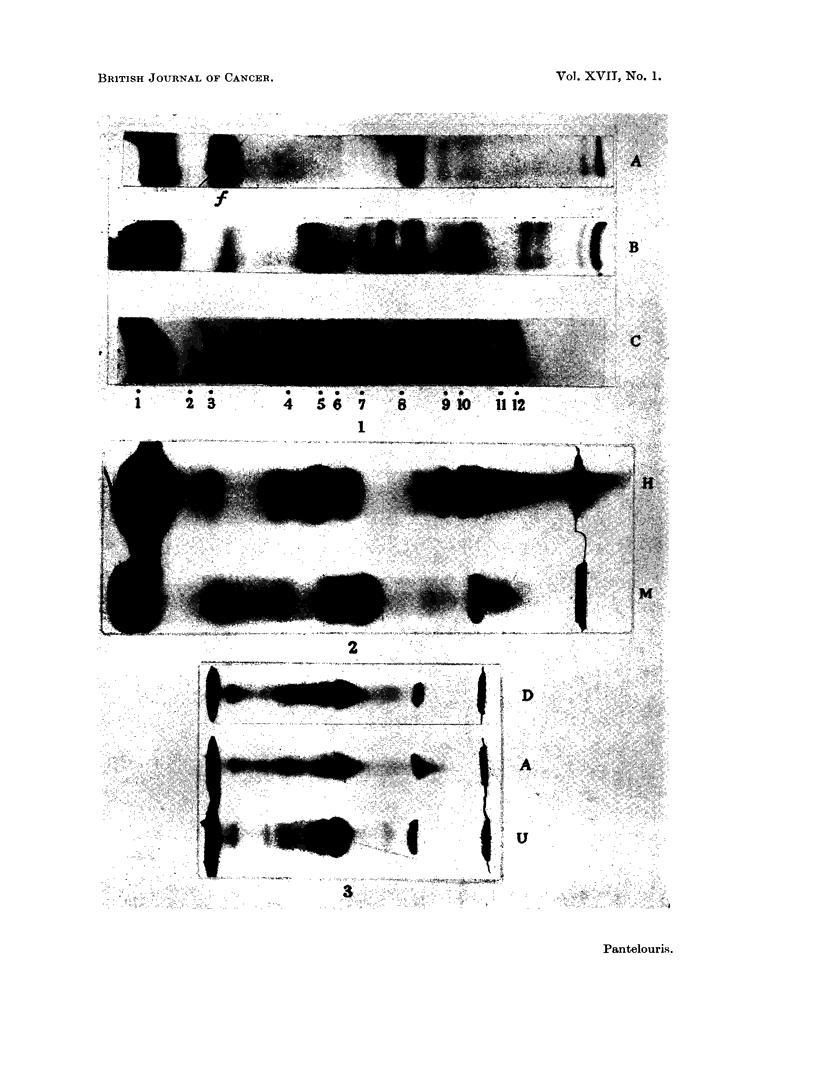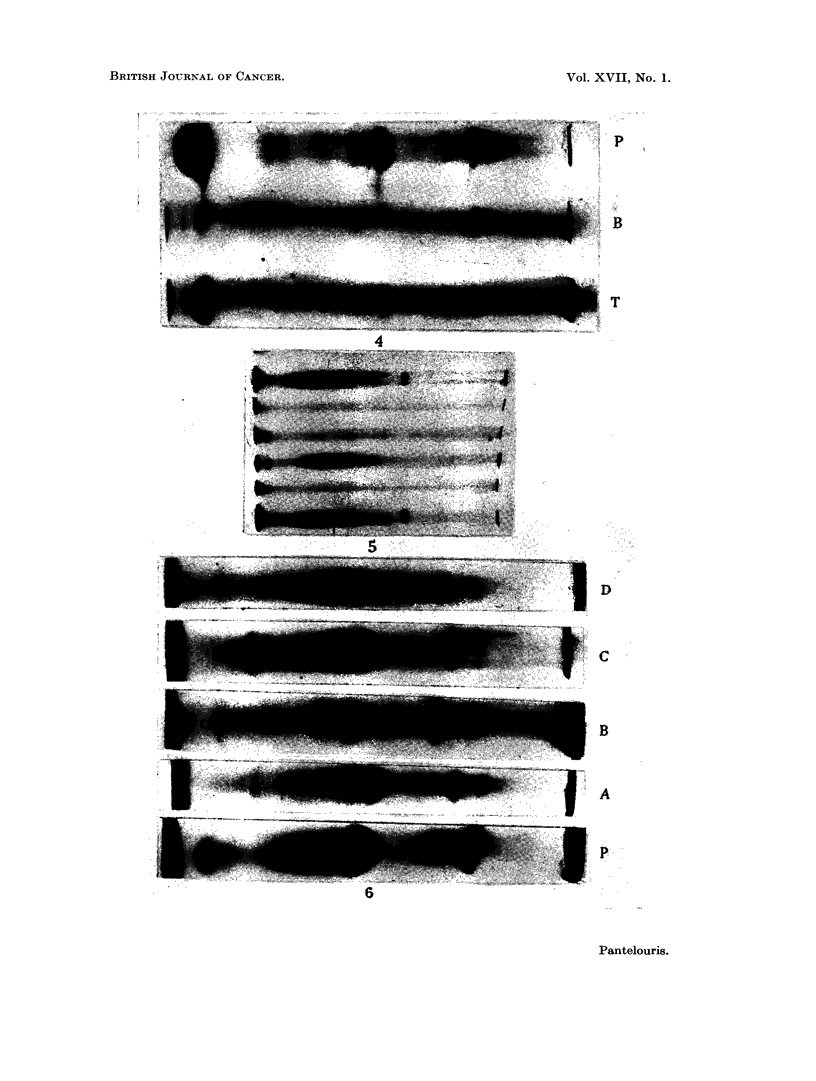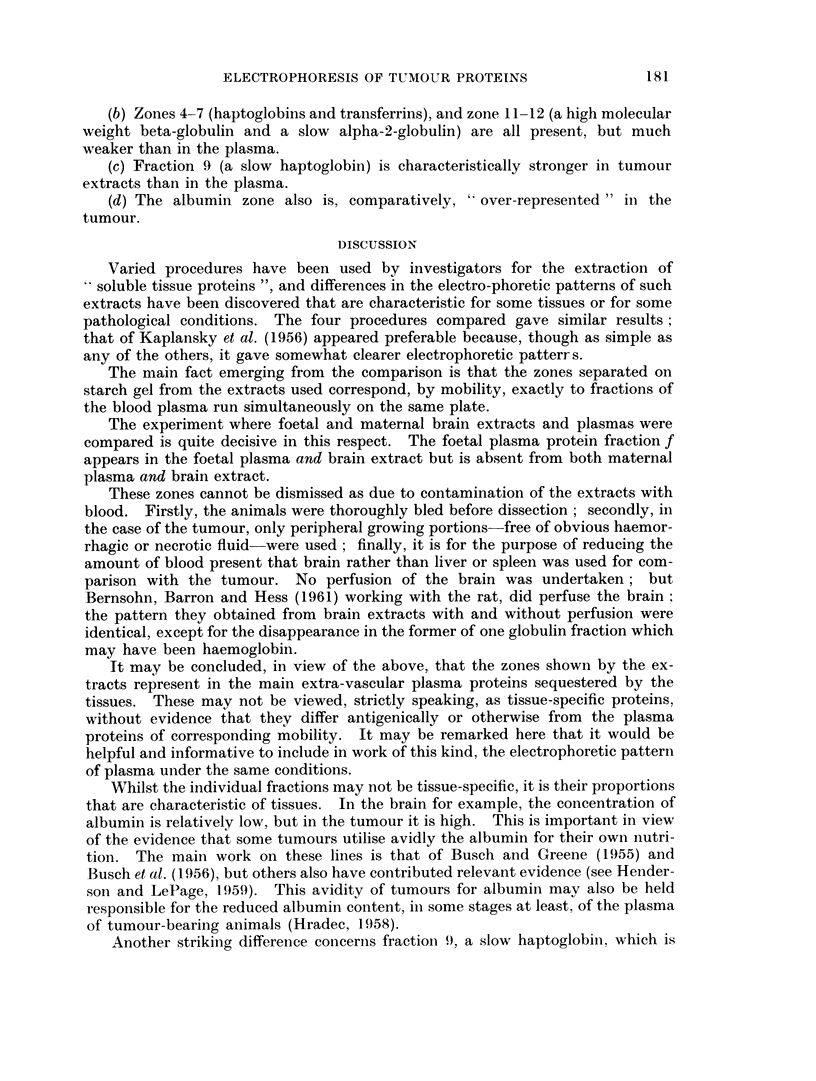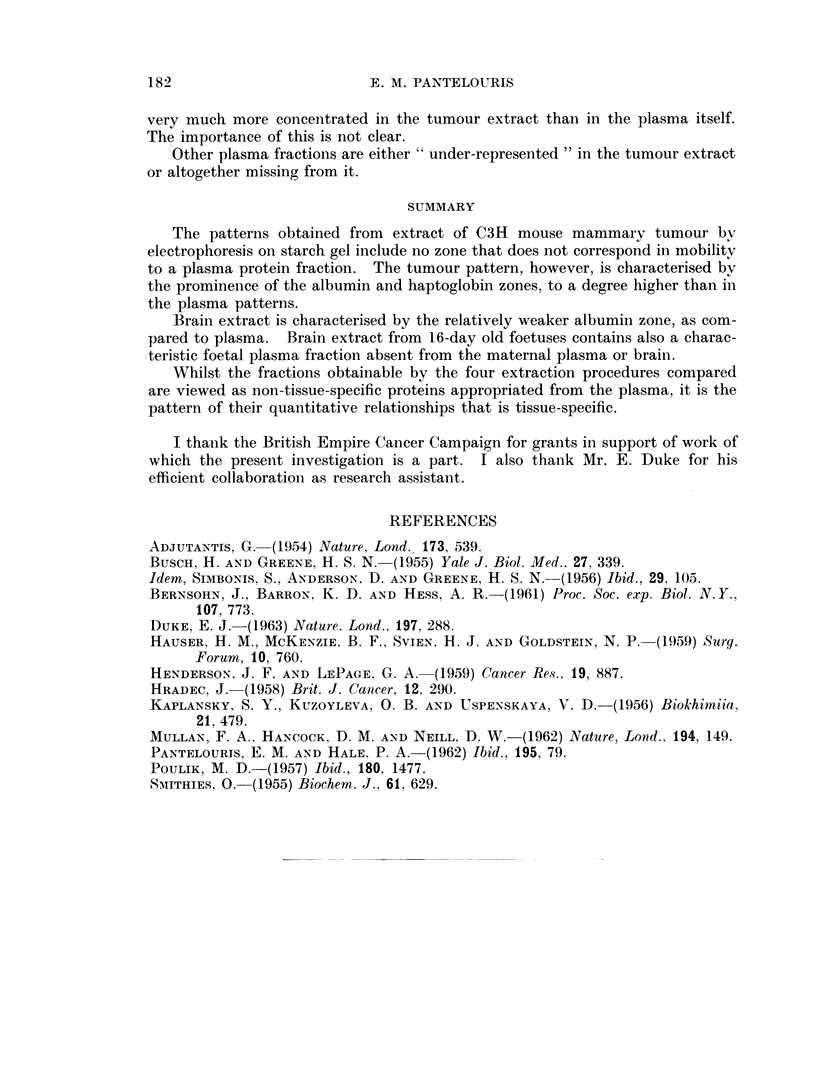# Starch Electrophoresis of Soluble Tumour Proteins

**DOI:** 10.1038/bjc.1963.25

**Published:** 1963-03

**Authors:** E. M. Pantelouris

## Abstract

**Images:**


### STARCH ELECTROPHORESIS OF SOLUBLE TUMOUR PROTEINS

E. M. PANTELOURIS

*From the Zoology Department, Queen's University, Belfast*

Received for publication December 1, 1962

In the work described here, extracts obtained (by four different procedures) from tumour and brain tissue of mice were compared electrophoretically with the protein fractions of blood plasma.

### MATERIALS AND METHODS

A mammary tumour spontaneously and frequently appearing in mice of strain C3H, and brain of the same mice were removed. Before dissection the animals were thoroughly bled under ether anaesthesia and the blood was used for the separation of plasma. The tissues were treated in the ways described by the following authors :

(A) Kaplansky, Kuzoyleva and Uspenskaya (1956), who were working with liver.

The tissue is homogenised in half its volume of physiological saline. Drops of ether are added and the tube is stirred and kept cool. When the contents become flaky and " thick ", the tube is centrifuged for 30 minutes at 2500 r.p.m. The clear bottom-layer is removed and warmed in a water-bath to 37° C. A spongy precipitate forms and is removed by centrifugation, leaving a clear supernatant to be used for the electrophoretic separation.

(B) Hauser *et al.* (1959), who were working with brain.

Homogenisation is here carried out in 0·25 M sucrose and is followed by centrifugation at 27,000 *g* for 2 hours.

(C) Mullan, Hancock and Neill (1962), working with liver.

Homogenisation in saline, centrifugation at 60,000 *g*, adjustment of the pH to 5·1 and further centrifugation at 3000 *g*.

(D) Adjutantis (1954), also working with liver.

The tissue is frozen and dried *in vacuo* and powdered ; it is further homogenised after suspension in isotonic sucrose solution and centrifuged to leave a clear supernatant.

As for the blood plasma, this was generally used untreated. For comparison, however, the plasma also was subjected to the same treatments as the tissues except, of course, for the homogenisation step, in some initial experiments.

*Technique of electrophoresis.*—Blood plasma and tissue extracts were run on starch plates as described by Smithies (1955), using the discontinuous buffer system developed by Poulik (1957). The voltage applied was 6 v/cm. and the run was continued for about 16 hours, with a current of 4 mA.

The starch plate was stained in Amido-black.

### RESULTS

### *Blood plasma*

Typically, mouse plasma gives rise on starch gel to about 12 fractions, excluding the $\gamma$-globulin region (Fig. 1). It will be noted that the plasma of foetuses, whilst weak in albumin, contains a very strong fraction (marked *f* in Fig. 1A) which is absent from the adult (Pantelouris and Hale, 1962).

Although the correspondence of most fractions may be deduced by visual comparison with human plasma (Fig. 2), a better definition has been worked out by Duke (1963).

Treatment of plasma by the four extraction procedures cited did not change its electrophoretic profile (Fig. 3), and untreated plasma was run parallel to the extracts in most experiments.

### *Brain*

The profiles given by the brain extracts were identical for all four procedures, but were perhaps somewhat clearer by method (A). Of course the extracts are dilute and hence the zones are weak and somewhat difficult to photograph (Fig. 4, B).

By mobility, all fractions seen correspond to zones in the plasma. The only differences are quantitative ; the most obvious is that the albumin fraction is weak in the brain extract as compared to plasma or to the tumour extract. The low albumin concentration in the two extracts results, incidentally, in the clear separation of three subfractions.

The exclusively prenatal fraction *f* appears in the foetal plasma as well as the foetal brain extract, but is absent from both plasma and brain extract of the mother (Fig. 5).

### *Tumour*

Typical patterns obtained from tumour extracts prepared by the four methods are given in Fig. 6. Again, there is no fraction in the tumour that is alien to the plasma. There are, however, striking differences in the relative density of various zones in plasma and tumour :

(*a*) Fraction 2 is absent from the tumour extract, but its neighbouring fraction 3 is present ; both are alpha-2 globulins.

---

### EXPLANATION OF PLATES

FIG. 1.—Starch-gel pherogram of mouse plasma. A, 16-day foetus of strain C3H, and B, the corresponding pregnant female. C, Adult female of strain A.

FIG. 2.—Comparison of human (H) and adult mouse plasma (M).

FIG. 3.—Pherograms of plasma after treatment by methods A and D, and untreated (U).

FIG. 4.—Pherogram of tumour (T) and brain (B) extracts and of plasma (P), the first obtained by method A.

FIG. 5.—Pherogram of plasma and brain extract. The two centre patterns correspond (from upper to lower) to brain extract and plasma of 16-day foetuses, and both show clearly the fraction *f*. The other four represent plasma and brain extract of the adult female and are devoid of fraction *f*.

FIG. 6.—Pherogram of plasmas (P) and of tumour extracts obtained by methods A, B, C and D as marked.

Pantelouris.

4

5

6

Pantelouris.

(*b*) Zones 4–7 (haptoglobins and transferrins), and zone 11–12 (a high molecular weight beta-globulin and a slow alpha-2-globulin) are all present, but much weaker than in the plasma.

(*c*) Fraction 9 (a slow haptoglobin) is characteristically stronger in tumour extracts than in the plasma.

(*d*) The albumin zone also is, comparatively, " over-represented " in the tumour.

### DISCUSSION

Varied procedures have been used by investigators for the extraction of " soluble tissue proteins ", and differences in the electro-phoretic patterns of such extracts have been discovered that are characteristic for some tissues or for some pathological conditions. The four procedures compared gave similar results ; that of Kaplansky *et al.* (1956) appeared preferable because, though as simple as any of the others, it gave somewhat clearer electrophoretic patterrs.

The main fact emerging from the comparison is that the zones separated on starch gel from the extracts used correspond, by mobility, exactly to fractions of the blood plasma run simultaneously on the same plate.

The experiment where foetal and maternal brain extracts and plasmas were compared is quite decisive in this respect. The foetal plasma protein fraction *f* appears in the foetal plasma *and* brain extract but is absent from both maternal plasma *and* brain extract.

These zones cannot be dismissed as due to contamination of the extracts with blood. Firstly, the animals were thoroughly bled before dissection ; secondly, in the case of the tumour, only peripheral growing portions—free of obvious haemorrhagic or necrotic fluid—were used ; finally, it is for the purpose of reducing the amount of blood present that brain rather than liver or spleen was used for comparison with the tumour. No perfusion of the brain was undertaken ; but Bernsohn, Barron and Hess (1961) working with the rat, did perfuse the brain ; the pattern they obtained from brain extracts with and without perfusion were identical, except for the disappearance in the former of one globulin fraction which may have been haemoglobin.

It may be concluded, in view of the above, that the zones shown by the extracts represent in the main extra-vascular plasma proteins sequestered by the tissues. These may not be viewed, strictly speaking, as tissue-specific proteins, without evidence that they differ antigenically or otherwise from the plasma proteins of corresponding mobility. It may be remarked here that it would be helpful and informative to include in work of this kind, the electrophoretic pattern of plasma under the same conditions.

Whilst the individual fractions may not be tissue-specific, it is their proportions that are characteristic of tissues. In the brain for example, the concentration of albumin is relatively low, but in the tumour it is high. This is important in view of the evidence that some tumours utilise avidly the albumin for their own nutrition. The main work on these lines is that of Busch and Greene (1955) and Busch *et al.* (1956), but others also have contributed relevant evidence (see Henderson and LePage, 1959). This avidity of tumours for albumin may also be held responsible for the reduced albumin content, in some stages at least, of the plasma of tumour-bearing animals (Hradec, 1958).

Another striking difference concerns fraction 9, a slow haptoglobin, which is very much more concentrated in the tumour extract than in the plasma itself. The importance of this is not clear.

Other plasma fractions are either " under-represented " in the tumour extract or altogether missing from it.

SUMMARY

The patterns obtained from extract of C3H mouse mammary tumour by electrophoresis on starch gel include no zone that does not correspond in mobility to a plasma protein fraction. The tumour pattern, however, is characterised by the prominence of the albumin and haptoglobin zones, to a degree higher than in the plasma patterns.

Brain extract is characterised by the relatively weaker albumin zone, as compared to plasma. Brain extract from 16-day old foetuses contains also a characteristic foetal plasma fraction absent from the maternal plasma or brain.

Whilst the fractions obtainable by the four extraction procedures compared are viewed as non-tissue-specific proteins appropriated from the plasma, it is the pattern of their quantitative relationships that is tissue-specific.

I thank the British Empire Cancer Campaign for grants in support of work of which the present investigation is a part. I also thank Mr. E. Duke for his efficient collaboration as research assistant.

### REFERENCES

ADJUTANTIS, G.—(1954) *Nature, Lond.*, **173**, 539.
BUSCH, H. AND GREENE, H. S. N.—(1955) *Yale J. Biol. Med.*, **27**, 339.
*Idem*, SIMBONIS, S., ANDERSON, D. AND GREENE, H. S. N.—(1956) *Ibid.*, **29**, 105.
BERNSOHN, J., BARRON, K. D. AND HESS, A. R.—(1961) *Proc. Soc. exp. Biol. N.Y.*, **107**, 773.
DUKE, E. J.—(1963) *Nature, Lond.*, **197**, 288.
HAUSER, H. M., MCKENZIE, B. F., SVIEN, H. J. AND GOLDSTEIN, N. P.—(1959) *Surg. Forum*, **10**, 760.
HENDERSON, J. F. AND LEPAGE, G. A.—(1959) *Cancer Res.*, **19**, 887.
HRADEC, J.—(1958) *Brit. J. Cancer*, **12**, 290.
KAPLANSKY, S. Y., KUZOYLEVA, O. B. AND USPENSKAYA, V. D.—(1956) *Biokhimiia*, **21**, 479.
MULLAN, F. A., HANCOCK, D. M. AND NEILL, D. W.—(1962) *Nature, Lond.*, **194**, 149.
PANTELOURIS, E. M. AND HALE, P. A.—(1962) *Ibid.*, **195**, 79.
POULIK, M. D.—(1957) *Ibid.*, **180**, 1477.
SMITHIES, O.—(1955) *Biochem. J.*, **61**, 629.